# Development and validation of a clinical and genetic model for predicting risk of severe COVID-19

**DOI:** 10.1017/S095026882100145X

**Published:** 2021-07-02

**Authors:** Gillian S. Dite, Nicholas M. Murphy, Richard Allman

**Affiliations:** Genetic Technologies Ltd, Fitzroy, Victoria, Australia

**Keywords:** Risk factors, risk prediction, severe COVID-19, single-nucleotide polymorphism

## Abstract

Clinical and genetic risk factors for severe coronavirus disease 2019 (COVID-19) are often considered independently and without knowledge of the magnitudes of their effects on risk. Using severe acute respiratory syndrome-coronavirus-2 (SARS-CoV-2) positive participants from the UK Biobank, we developed and validated a clinical and genetic model to predict risk of severe COVID-19. We used multivariable logistic regression on a 70% training dataset and used the remaining 30% for validation. We also validated a previously published prototype model. In the validation dataset, our new model was associated with severe COVID-19 (odds ratio per quintile of risk = 1.77, 95% confidence interval (CI) 1.64–1.90) and had acceptable discrimination (area under the receiver operating characteristic curve = 0.732, 95% CI 0.708–0.756). We assessed calibration using logistic regression of the log odds of the risk score, and the new model showed no evidence of over- or under-estimation of risk (*α* = −0.08; 95% CI −0.21−0.05) and no evidence or over-or under-dispersion of risk (*β* = 0.90, 95% CI 0.80–1.00). Accurate prediction of individual risk is possible and will be important in regions where vaccines are not widely available or where people refuse or are disqualified from vaccination, especially given uncertainty about the extent of infection transmission among vaccinated people and the emergence of SARS-CoV-2 variants of concern.

## Introduction

The coronavirus disease 2019 (COVID-19) pandemic continues to dominate global public health, with countries having varying success with infection control measures and social distancing protocols [1], coupled with this are the logistical challenges with the distribution of vaccines [2] and the emergence of severe acute respiratory syndrome-coronavirus-2 (SARS-CoV-2) variants of concern [3, 4]. Of those who become infected with SARS-CoV-2, 10%–15% will develop severe COVID-19 requiring hospitalisation and 5% will require intensive care [5]. At all stages of the pandemic, there has been an urgent need for accurate quantification of risk of severe COVID-19 to inform protection from infection for those at increased risk.

Epidemiological analyses have recognised that male sex and increasing age are risk factors for severe COVID-19 and that common medical comorbidities contribute to individual risk [6–8]. Our previous analysis showed that the effects of sex and age are attenuated when comorbidities are taken into account [9]. The effect of human genetic variation on COVID-19 severity has been examined by the COVID-19 Host Genetics Initiative, which has now released several meta-analyses of the available genome-wide association studies of COVID-19 severity [10, 11]. Using population controls, Ellinghaus *et al*. [12] identified two loci (3p21.31 and 9q34.2) as being strongly associated with respiratory failure from COVID-19 and Shelton *et al*. [13] identified the 3p21.31 locus as being associated with severe COVID-19. Also using population controls, Pairo-Castineira *et al*. [14] identified eight single-nucleotide polymorphisms (SNPs) that achieved genome-wide significance for intensive care admission and identified six SNPs (two of which were also in the panel of eight SNPs) associated with risk of hospitalisation.

The emergency authorisation of SARS-CoV-2 vaccines [15] does not diminish the value of accurate prediction of individual risk of severe COVID-19. Extensive vaccine disqualification criteria (such as pre-existing conditions, pregnancy and age), vaccine hesitancy, uncertainty as to whether the vaccines are effective against emerging variants of concern [4] and an unknown extent to which vaccines prevent the transmission of infection mean that many people will be at risk of severe COVID-19 should they become infected with SARS-CoV-2.

We previously developed a prototype risk model [9] based upon early data from the UK Biobank [16, 17] and SNPs identified from the COVID-19 Host Genetics Initiative Release 2 meta-analysis of hospitalised *vs.* non-hospitalised COVID-19 cases (which was at that time almost exclusively UK Biobank samples) [10, 18]. Our prototype model appeared to perform well but was based on a small sample size from the first wave of the pandemic [9]. We decided not to attempt validation in that dataset because of our concern about the representativeness of the data (the SARS-CoV-2 testing data was ascertained early in the pandemic when the limitations on testing availability in the United Kingdom meant that mild and asymptomatic cases were not identified) and because Release 2 results from the COVID-19 Host Genetics Initiative were predominately from UK Biobank samples.

In the interim, the UK Biobank has released further data from participants confirmed to be infected with SARS-CoV-2. This latest data release (2205 cases and 5416 controls) has a larger proportion of non-hospitalised people, providing more confidence that they are more representative. In this paper, we perform a validation study of our prototype model and take advantage of the larger dataset that is now available to develop and validate a new clinical and genetic model to predict risk of severe COVID-19.

## Methods

### UK Biobank data and eligibility

Since our first paper on the development of a risk prediction model for severe COVID-19 [9], the UK Biobank [16, 17] has accumulated a large number of additional SARS-CoV-2 test results [19]. For this analysis, we downloaded an updated results file on 8 January 2021. As in our first paper, eligible participants were active UK Biobank participants with a positive SARS-CoV-2 test result and who had SNP and hospital data available [9]. Of the 47 990 UK Biobank participants with at least one SARS-CoV-2 test result, 8672 (18.1%) had a positive test result, and of these, 7621 met our eligibility criteria.

As we did previously [9], we used source of test result as a proxy for severity of disease, where inpatient results were considered severe disease (cases) and outpatient results were considered non-severe disease (controls). If a participant had more than one test result, we classified them as having severe disease if at least one of their results was from an inpatient setting. Of the 7621 eligible participants, 2205 (28.9%) were cases and 5416 (71.7%) were controls.

### Data extraction

We used UK Biobank clinical and genetic data that we had previously downloaded (see Table 1). We used Plink version 1.9 [20, 21] to extract SNP data from the UK Biobank imputation dataset. We extracted genotypes of the 64 SNPs that were used to calculate the SNP score in our prototype model [9] and the 12 SNPs from Pairo-Castineira *et al*. [14] We also identified 43 SNPs from the B1_ALL (hospitalised *vs.* non-hospitalised cases of COVID-19) results of the COVID19-hg GWAS meta-analyses round 4, conducted by the COVID-19 Host Genetics Initiative consortium [10, 22]. These SNPs were selected by pruning variants with a *P* value of greater than 10^−5^ and linkage disequilibrium variants that had an *R*^2^ of greater than 0.5 for all populations. Of these 43 SNPS, 40 were available for extraction in the UK Biobank imputation dataset. The SNPs considered in the current paper are listed in Supplementary Table S1.

**Table 1.** Characteristics of cases and controls in the training and validation datasets for the variables considered for inclusion in the new model

	Training		Validation	
Variable	Cases *N* = 1544	Controls *N* = 3791	Cases *N* = 661	Controls *N* = 1625
	**Mean (s.d.)**	**Mean (s.d.)**	**Mean (s.d.)**	**Mean (s.d.)**
Inverse of BMI				
10/(kg/m^2^)	0.35 (0.06)	0.37 (0.06)	0.35 (0.06)	0.36 (0.06)
	***N*** (**%)**	***N*** (**%)**	***N*** (**%)**	***N*** (**%)**
Age group (years)				
50–54	97 (6.3)	465 (12.3)	40 (6.1)	192 (11.8)
55–59	178 (11.5)	872 (23.0)	85 (12.9)	401 (24.7)
60–64	144 (9.3)	668 (17.6)	70 (10.6)	290 (17.9)
65–69	197 (12.8)	578 (15.3)	83 (12.6)	240 (14.8)
70–74	343 (22.2)	589 (15.5)	127 (19.2)	247 (15.2)
75–79	436 (28.2)	481 (12.7)	190 (28.7)	196 (12.1)
80+	149 (9.7)	138 (3.6)	66 (10.0)	59 (3.6)
Sex				
Female	665 (43.1)	2080 (54.9)	281 (42.5)	857 (52.7)
Male	879 (56.9)	1711 (45.1)	380 (57.5)	768 (47.3)
Ethnicity				
White	1381 (89.4)	3481 (91.8)	599 (90.6)	1486 (91.5)
Other/Unknown	163 (10.6)	310 (8.2)	62 (9.4)	139 (8.6)
ABO blood type				
O	627 (40.6)	1472 (38.8)	300 (45.4)	640 (39.4)
A	701 (45.4)	1764 (46.5)	265 (40.1)	750 (46.2)
B	164 (10.6)	393 (10.4)	70 (10.6)	169 (10.4)
AB	52 (3.4)	162 (4.3)	26 (3.9)	66 (4.1)
Asthma				
No	1286 (83.3)	3355 (88.5)	549 (83.1)	1403 (86.3)
Yes	258 (16.7)	436 (11.5)	112 (16.9)	222 (13.7)
Autoimmune disease (rheumatoid arthritis, lupus or psoriasis)				
No	1448 (93.8)	3654 (96.4)	616 (93.2)	1571 (96.7)
Yes	96 (6.2)	137 (3.6)	45 (6.8)	54 (3.3)
Cancer – haematological				
No	1494 (96.8)	3765 (99.3)	637 (96.4)	1615 (99.4)
Yes	50 (3.2)	26 (0.7)	24 (3.6)	10 (0.6)
Cancer – non-haematological				
No	1217 (78.8)	3323 (87.7)	525 (79.4)	1425 (87.7)
Yes	327 (21.2)	468 (12.4)	136 (20.6)	200 (12.3)
Cerebrovascular disease				
No	1338 (86.7)	3626 (95.7)	565 (85.5)	1555 (95.7)
Yes	206 (13.3)	165 (4.4)	96 (14.5)	70 (4.3)
Diabetes				
No	1168 (75.7)	3453 (91.1)	525 (79.4)	1470 (90.5)
Yes	376 (24.4)	338 (8.9)	136 (20.6)	155 (9.5)
	***N*** (**%)**	***N*** (**%)**	***N*** (**%)**	***N*** (**%)**
Heart disease				
No	1013 (65.6)	3205 (84.5)	454 (68.7)	1374 (84.6)
Yes	531 (34.4)	586 (15.5)	207 (31.3)	251 (15.5)
Hypertension				
No	679 (44.0)	2661 (70.2)	304 (46.0)	1134 (69.8)
Yes	865 (56.0)	1130 (29.8)	357 (54.0)	491 (30.2)
Immunocompromised				
No	1525 (98.8)	3780 (99.7)	653 (98.8)	1620 (99.7)
Yes	19 (1.2)	11 (0.3)	8 (1.2)	5 (0.3)
Kidney disease				
No	1318 (85.4)	3677 (97.0)	581 (87.9)	1562 (96.1)
Yes	226 (14.6)	114 (3.0)	80 (12.1)	63 (3.9)
Liver disease				
No	1442 (93.4)	3683 (97.2)	613 (92.7)	1579 (97.2)
Yes	102 (6.6)	108 (2.9)	48 (7.3)	46 (2.8)
Respiratory disease (excluding asthma)				
No	1026 (66.5)	3487 (92.0)	448 (67.8)	1489 (91.6)
Yes	518 (33.6)	304 (8.0)	213 (32.2)	136 (8.4)
rs112641600				
C/C	1249 (80.9)	2972 (78.4)	525 (79.4)	1271 (78.2)
T/C	262 (17.0)	708 (18.7)	120 (18.2)	317 (19.5)
T/T	11 (0.7)	58 (1.5)	5 (0.76)	20 (1.2)
Missing	22 (1.4)	53 (1.4)	11 (1.7)	17 (1.1)
rs10755709				
A/A	708 (45.9)	1824 (48.1)	300 (45.4)	749 (46.1)
G/A	618 (40.0)	1535 (40.5)	291 (44.0)	701 (43.1)
G/G	169 (11.0)	332 (8.8)	58 (8.8)	124 (7.6)
Missing	49 (3.2)	100 (2.6)	12 (1.8)	51 (3.1)
rs16873740				
T/T	1171 (75.8)	2972 (78.4)	495 (74.9)	1266 (77.9)
A/T	340 (22.0)	763 (20.1)	157 (23.8)	336 (20.7)
A/A	32 (2.1)	52 (1.4)	8 (1.2)	20 (1.2)
Missing	1 (0.1)	4 (0.1)	1 (0.2)	3 (0.2)
rs118072448				
T/T	1346 (87.2)	3215 (84.8)	581 (87.9)	1380 (84.9)
C/T	188 (12.2)	536 (14.1)	71 (10.7)	231 (14.2)
C/C	10 (0.7)	40 (1.1)	9 (1.4)	14 (0.9)
Missing	0 (0.0)	0 (0.0)	0 (0.0)	0 (0.0)
	***N*** (**%)**	***N*** (**%)**	***N*** (**%)**	***N*** (**%)**
rs7027911				
G/G	367 (23.8)	1023 (27.0)	156 (23.6)	398 (24.5)
A/G	606 (39.3)	1415 (37.3)	261 (39.5)	676 (41.6)
A/A	240 (15.5)	553 (14.6)	111 (16.8)	229 (14.1)
Missing	331 (21.4)	800 (21.1)	133 (20.1)	322 (19.8)
rs71481792				
A/A	239 (15.5)	514 (13.6)	122 (18.5)	246 (15.1)
T/A	701 (45.4)	1704 (45.0)	263 (39.8)	712 (43.8)
T/T	522 (33.8)	1416 (37.4)	247 (37.4)	591 (36.4)
Missing	82 (5.3)	157 (4.1)	29 (4.4)	76 (4.7)
rs1984162				
A/A	827 (53.6)	2144 (56.6)	363 (54.9)	865 (53.2)
G/A	612 (39.6)	1416 (37.4)	249 (37.7)	642 (39.5)
G/G	105 (6.8)	231 (6.1)	49 (7.4)	118 (7.3)
Missing	0 (0.0)	0 (0.0)	0 (0.0)	0 (0.0)
rs115492982				
G/G	1529 (99.0)	3774 (99.6)	654 (98.9)	1621 (99.8)
A/G	14 (0.9)	15 (0.4)	7 (1.1)	3 (0.2)
A/A	1 (0.1)	0 (0.0)	0 (0.0)	0 (0.0)
Missing	0 (0.0)	2 (0.1)	0 (0.0)	1 (0.1)
rs112317747				
T/T	1410 (91.3)	3518 (92.8)	610 (92.3)	1521 (93.6)
C/T	115 (7.5)	236 (6.2)	44 (6.7)	87 (5.4)
C/C	2 (0.1)	0 (0.0)	0 (0.0)	0 (0.0)
Missing	17 (1.1)	37 (1.0)	7 (1.1)	17 (1.1)
rs2034831				
A/A	1284 (83.2)	3242 (85.5)	550 (83.2)	1375 (84.6)
C/A	200 (13.0)	399 (10.5)	80 (12.1)	190 (11.7)
C/C	8 (0.5)	18 (0.5)	10 (1.5)	8 (0.5)
Missing	52 (3.4)	132 (3.5)	21 (3.2)	52 (3.2)
rs35896106				
C/C	1251 (81.0)	3166 (83.5)	537 (81.2)	1373 (84.5)
T/C	231 (15.0)	514 (13.6)	104 (15.7)	203 (12.5)
C/C	13 (0.8)	23 (0.6)	5 (0.8)	12 (0.7)
Missing	49 (3.2)	88 (2.3)	15 (2.3)	37 (2.3)
rs76374459				
G/G	1318 (85.4)	3320 (87.6)	567 (85.8)	1440 (88.6)
C/G	187 (12.1)	394 (10.4)	83 (12.6)	150 (9.2)
C/C	9 (0.6)	12 (0.3)	1 (0.2)	8 (0.5)
Missing	30 (1.9)	65 (1.7)	10 (1.5)	27 (1.7)
	***N*** (**%)**	***N*** (**%)**	***N*** (**%)**	***N*** (**%)**
rs35652899				
C/C	1286 (83.3)	3236 (85.4)	553 (83.7)	1406 (86.5)
G/C	222 (14.4)	493 (13.0)	97 (14.7)	187 (11.5)
G/G	14 (0.9)	20 (0.5)	3 (0.5)	10 (0.6)
Missing	22 (1.4)	42 (1.1)	8 (1.2)	22 (1.4)
rs76488148				
G/G	1385 (89.7)	3463 (91.4)	603 (91.2)	1488 (91.6)
T/G	144 (9.3)	290 (7.7)	49 (7.4)	119 (7.3)
T/T	5 (0.3)	7 (0.2)	0 (0.0)	4 (0.3)
Missing	10 (0.7)	31 (0.8)	9 (1.4)	14 (0.9)

s.d., standard deviation.

### Validation of prototype model

For the validation of our prototype risk model [9], we used the 1234 cases and 4805 controls that were not included in our previous paper. We constructed relative risk scores for both the clinical model and the combined clinical and SNP score model using the exponent of the sum of the intercept and the beta coefficients for each risk factor in the prototype model [9].

### Development and validation of the new model

To develop a new model to predict risk of severe COVID-19, we used all of the available data and randomly divided it into a 70% training dataset and a 30% validation dataset (ensuring that the datasets were balanced for case and control status). We used multiple imputation with 20 imputations to address the missing data for body mass index (BMI) (linear regression) and the SNP data (predictive mean matching) for the development of the new model in the training dataset. To more closely reflect the availability of data in the real world, we did not use imputed data in the validation dataset.

The clinical variables considered for inclusion in the new model were age, sex, BMI, ethnicity (Caucasian *vs* other), ABO blood type and the following chronic health conditions: asthma, autoimmune disease (rheumatoid arthritis, lupus or psoriasis), haematological cancer, non-haematological cancer, cerebrovascular disease, diabetes, heart disease, hypertension, immunocompromised, kidney disease, liver disease and respiratory disease (excluding asthma). Dummy variables were used for the categorical classifications of age and ABO blood type.

The SNPs selected for consideration in the development of the new model came from three sources: (i) the 64 SNPs from our prototype model [9], which include 62 SNPs from the results of the COVID-19 Host Genetics Initiative's COVID19 Round 2 meta-analysis of non-hospitalised *vs.* hospitalised cases of COVID-19 and the two SNPs from Ellinghaus *et al*. [12]; (ii) the 12 SNPs from Pairo-Castineira *et al*. [14]; and (iii) the 40 SNPs newly selected from the results of the COVID-19 Host Genetics Initiative's COVID19 Round 4 meta-analysis of non-hospitalised *vs.* hospitalised cases of COVID-19 [10, 22]. To avoid reliance on potentially inaccurate summary statistics to construct a polygenic risk score, we used unadjusted logistic regression in the multiple imputation training dataset to identify the subset of SNPs that were associated with risk of severe COVID-19 with *P* < 0.05 (see Supplementary Table S1) and used these as individual risk factors (with a per allele effect) to build our new model.

### Statistical methods

#### Development of new model

We used multivariable logistic regression in the multiple imputation training dataset to develop the new model to predict risk of severe COVID-19. We began with a model that included all of the clinical variables and the SNPs with unadjusted associations with severe COVID-19. We then used backwards stepwise selection to develop the most parsimonious model. For the removed variables, we made a final determination on their inclusion or exclusion by adding them one at a time to the parsimonious model. To directly compare the effect sizes of the variables in the final model, regardless of the scale on which they were measured, we used the odds per adjusted standard deviation [23]. We used the intercept and beta coefficients from the new model to calculate the COVID-19 risk score (as a % risk) for all eligible UK Biobank participants.

#### Model performance

The association between risk score and severe COVID-19 was assessed using logistic regression to estimate the OR per quintile of risk score. We assessed model discrimination using the area under the receiver operating characteristic curve (AUC). Where warranted, we plotted the receiver operating characteristic curve of the model.

We assessed calibration using logistic regression of the log odds of the risk score to estimate the intercept and the slope (beta coefficient). An intercept close to 0 indicates good calibration, while an intercept of less than 0 indicates overall overestimation and an intercept of greater than 0 indicates overall underestimation of risk.

In terms of the dispersion of the risk score, a slope of close to 1 indicates good estimation across the spectrum of risk. A slope of less than 1 means that the predicted probabilities do not vary enough (i.e. underestimation of true high risk and overestimation of true low risk). Conversely, a slope of greater than one means that the predicted probabilities vary too much (i.e. underestimation of true low risk and overestimation of true high risk). Where helpful, we also used a calibration plot to illustrate the fit of a model.

We used Stata (version 16.1) [24] for analyses; all statistical tests were two-sided and *P* < 0.05 was considered nominally statistically significant.

### Ethics approval

The UK Biobank has Research Tissue Bank approval (REC #11/NW/0382) that covers analysis of data by approved researchers. All participants provided written informed consent to the UK Biobank before data collection began. This research has been conducted using the UK Biobank resource under Application Number 47401.

### Data availability statement

The data underlying this article was provided by the UK Biobank and we do not have permission to share the data. Researchers wishing to access the data used in this study can apply directly to the UK Biobank at https://www.ukbiobank.ac.uk/register-apply/. Stata 16.1 code for the analysis is available from the corresponding author on request.

## Results

In the results file downloaded on 8 January 2021, there were 2205 eligible cases with severe COVID-19 and 5416 eligible controls with non-severe COVID-19.

### Validation of prototype model

Characteristics of the new UK Biobank participants (1234 cases and 4805 controls) with positive SARS-CoV-2 test results are shown in Supplementary Table S2.

The odds ratio (OR) per quintile showed that the clinical risk score was strongly associated with severe COVID-19 (OR 1.70; 95% confidence interval (CI) 1.62–1.79; *P* < 0.001) and that the combined clinical and SNP risk score was less strongly associated with severe COVID-19 (OR 1.45; 95% CI 1.38–1.52; *P* < 0.001); there was no association with severe COVID-19 for the SNP score (OR 0.98; 95% CI 0.94–1.03; *P* = 0.5). The discrimination of cases and controls was acceptable for the clinical score (AUC = 0.711; 95% CI 0.694–0.727), lower for the combined clinical and SNP score (AUC = 0.657; 95% CI 0.639–0.674) and poor for the SNP score alone (AUC = 0.491; 95% CI 0.473–0.509).

Assessment of model calibration showed that overall, risk was overestimated for both the clinical risk model (*α* = −1.72; 95% CI −1.80 to −1.65; *P* < 0.001) and the clinical and SNP model (*α* = −1.63; 95% CI −1.71 to −1.54; *P* < 0.001). For the clinical model, there was no evidence of poor dispersion (*β* = 1.03, 95% CI 0.94–1.12, *P* = 0.5), while the predictions of the combined clinical and SNP model varied too much (*β* = 0.59, 95% CI 0.52–0.65, *P* < 0.001).

### Development and validation of the new model

Table 1 shows the characteristics of the 1544 cases and 3791 controls in the 70% training dataset and the 661 cases and 1625 controls in the 30% validation data set. In the training dataset, the mean age was 69.8 years (s.d. = 8.6) for cases and 64.6 years (s.d. = 8.4) for controls, and the mean BMI was 29.3 kg/m^2^ (s.d. = 5.3) for cases and 28.0 kg/m^2^ (s.d. = 4.9) for controls. In the validation dataset, the mean age was 69.7 years (s.d. = 8.7) for cases and 64.4 years (s.d. = 8.4) for controls, and the mean BMI was 29.4 kg/m^2^ (s.d. = 5.6) for cases and 28.3 kg/m^2^ (s.d. = 5.0) for controls.

#### Training

In the age and sex model, being male and being in one of the four older age groups conferred a substantially increased risk of severe COVID-19 (Table 2), with an OR 1.60 for being male and ORs ranging from 2.74 for the age groups from 65–69 years to 4.95 for the 80+ years group. Direct comparison of the effect size of each variable showed that the age group 75–79 years was the strongest risk factor (with odds per adjusted standard deviation of 1.58), followed by the 70–74 and 80–84 groups (with odds per adjusted standard deviations of 1.42 and 1.34, respectively).

**Table 2.** Adjusted ORs and odds per adjusted standard deviation for the risk factors in the age and sex model for risk of severe COVID-19 in the training dataset and adjusted ORs in the validation dataset

	Training dataset					Validation dataset		
Variable	Adjusted OR	95% CI	*P* value	Odds per adjusted standard deviation	95% CI	Adjusted OR	95% CI	*P* value
Age group (years)								
65–69	1.60	1.32–1.94	<0.001	1.18	1.10–1.26	0.84	0.62–1.14	0.3
70–74	2.74	2.31–3.24	<0.001	1.42	1.34–1.50	1.19	0.88–1.60	0.3
75–79	4.20	3.55–4.97	<0.001	1.58	1.50–1.67	1.72	1.32–2.25	<0.001
80+	4.95	3.83–6.39	<0.001	1.34	1.28–1.41	3.24	2.50–4.19	<0.001
Sex								
Male	1.48	1.31–1.67	<0.001	1.21	1.14–1.29	1.41	1.17–1.70	<0.001

*Note*: Adjusted OR and odds per adjusted standard deviation calculated using the original dataset because there was no missing data in this model.

**Fig. 1.**
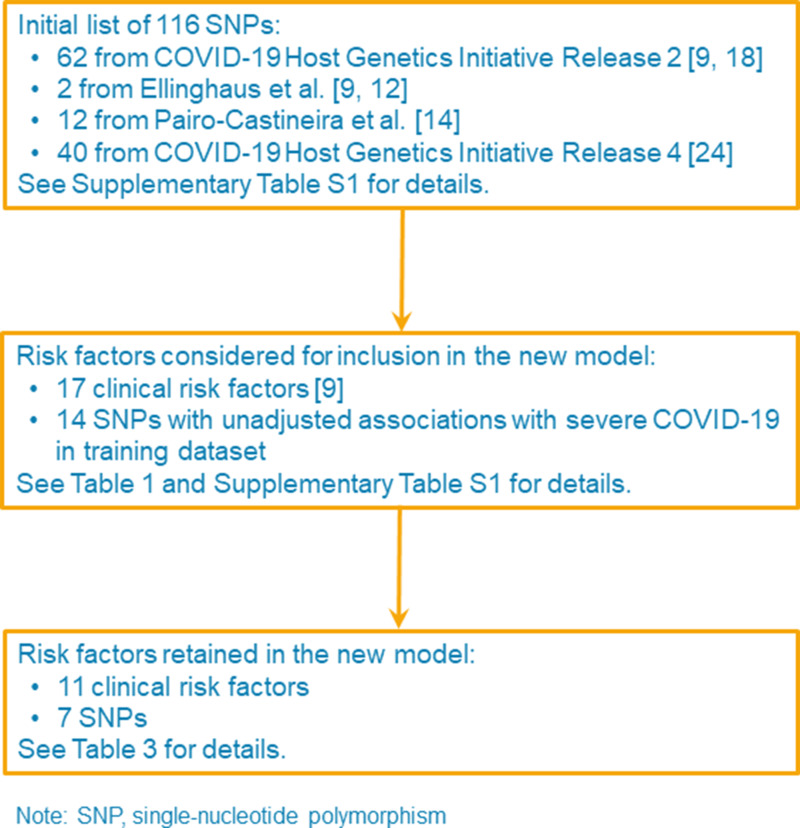
Flow chart of risk factor selection for the development of the new model in the training dataset.

The new model was developed from the variables in Table 1, which include the clinical variables and the 14 SNPs identified as having unadjusted per allele ORs with *P*-values < 0.05 (see Supplementary Table S1). The variables retained in the new model are shown in Table 3 and comprise three age groups (70–74, 75–79 and 80–84 years), sex, ethnicity, BMI, six comorbidities and seven SNPs (see Fig. 1 for a flow chart of variable selection). Compared with the age and sex model, the effects of sex and age group were attenuated in the new model, with an OR 1.27 for being male, the 70–74 years age group not being at increased risk, and ORs for the other age groups ranging from 1.77 for the 70–74 years group to 2.76 for the 80+ years group.

**Table 3.** Adjusted ORs and odds per adjusted standard deviation for the risk factors in the new model for risk of severe COVID-19 in the training dataset and adjusted ORs in the validation dataset

	Training dataset					Validation dataset		
Variable	Adjusted OR	95% CI	*P* value	Odds per adjusted standard deviation	95% CI	Adjusted OR	95% CI	*P* value
Age group (years)								
70–74	1.77	1.49–2.12	<0.001	1.22	1.15, 1.30	1.33	0.93, 1.91	0.1
75–79	2.28	1.90–2.73	<0.001	1.29	1.22, 1.36	1.26	0.90, 1.77	0.2
80+	2.76	2.09–3.64	<0.001	1.20	1.14–1.26	2.04	1.48–2.81	<0.001
Sex								
Male	1.27	1.12–1.46	<0.001	1.13	1.06–1.20	1.32	1.04–1.68	0.02
Ethnicity								
Non-white	1.34	1.06–1.70	0.02	1.08	1.01–1.14	1.14	0.67–1.95	0.6
Inverse of BMI								
10/(kg/m^2^)	0.20	0.06–0.66	0.008	0.91	0.85–0.97	0.06	0.01–0.50	0.01
Cancer – haematological								
Yes	2.73	1.62–4.60	<0.001	1.09	1.04–1.13	2.57	1.12–5.89	0.03
Cancer – non-haematological								
Yes	1.29	1.08–1.54	0.005	1.09	1.03–1.15	1.11	0.81–1.53	0.5
Cerebrovascular disease								
Yes	1.50	1.17–1.92	0.001	1.08	1.03–1.14	2.13	1.38–3.30	0.001
Diabetes								
Yes	1.54	1.26–1.87	<0.001	1.12	1.06–1.18	1.34	0.94–1.91	0.1
Hypertension								
Yes	1.34	1.15–1.56	<0.001	1.12	1.06–1.19	1.54	1.18–2.01	0.06
Kidney disease								
Yes	2.00	1.53–2.61	<0.001	1.12	1.07–1.17	1.59	0.99–2.56	<0.001
Respiratory disease (excluding asthma)								
Yes	3.23	2.71–3.85	<0.001	1.35	1.29–1.42	3.22	2.36–4.40	<0.001
rs112641600								
Per T allele	0.79	0.68–0.92	0.003	0.90	0.84–0.97	0.98	0.74–1.28	0.9
rs10755709								
Per G allele	1.13	1.02–1.25	0.02	1.09	1.02–1.16	1.03	0.85–1.24	0.8
rs118072448								
Per C allele	0.82	0.69–0.98	0.03	0.93	0.86–0.99	0.91	0.66–1.24	0.5
rs7027911								
Per A allele	1.11	1.00–1.23	0.05	1.07	1.00–1.15	1.15	0.96–1.36	0.1
rs71481792								
Per T allele	0.90	0.82–1.00	0.04	0.93	0.87–0.99	0.95	0.80–1.13	0.6
rs112317747								
Per C allele	1.31	1.02–1.70	0.04	1.06	1.00–1.13	1.42	0.88–2.30	0.2
rs2034831								
Per C allele	1.27	1.05–1.53	0.01	1.08	1.02–1.15	1.30	0.95–1.77	0.1

*Note*: Training dataset analyses used multiple imputation data; odds per adjusted standard deviation calculated using only the first imputation dataset. Validation dataset analyses used non-imputed data.

Direct comparison of the effect size of each variable showed that respiratory disease was the strongest risk factor with odds per adjusted standard deviation of 1.35, followed by the three older age groups with odds per adjusted standard deviations of 1.20–1.29. The other clinical risk factors and SNPs had odds per adjusted standard deviation in the range 1.07–1.13 (or the equivalent protective effect).

The age and sex model had good discrimination of cases and controls with an AUC of 0.676 (95% CI 0.659–0.692) but the new model with an AUC of 0.752 (95% CI 0.737–0.767) was a substantial improvement (*χ*^2^ = 149.40, df = 1, *P* < 0.001).

#### Validation

In the non-imputed validation dataset, the age and sex model and the new model were associated with severe COVID-19. The OR per quintile for the age and sex model was 1.49 (95% CI 1.40–1.59; *P* < 0.001), while the new model had a substantially higher OR per quintile of 1.77 (95% CI 1.64–1.90; *P* < 0.001). The ORs for the variables in the age and sex model and in the new model in the validation dataset are shown in Tables 2 and 3, respectively.

**Fig. 2.**
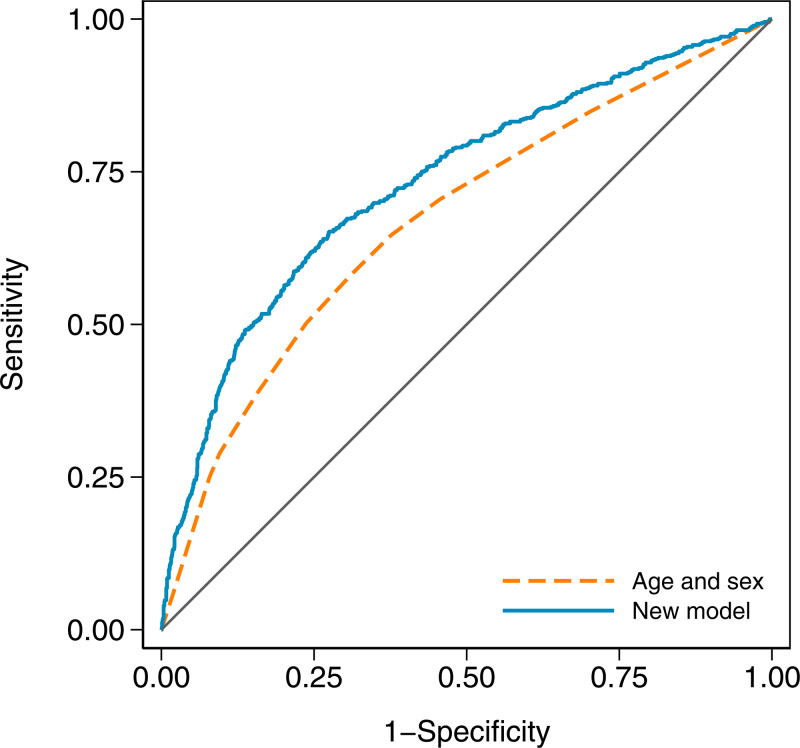
Receiver operating characteristic curves for the age and sex model and the new model in the validation dataset. The new model has an area under the curve (AUC) of 0.732 (95% CI 0.708–0.756), and the age and sex model has an AUC of 0.671 (95% CI 0.646–0.696).

In terms of discrimination between cases and controls, the age and sex model had an AUC of 0.671 (95% CI 0.646–0.696), while the new model with an AUC of 0.732 (95% CI 0.708–0.756) was a substantial improvement (*χ*^2^ = 41.23, df = 1, *P* < 0.001). The receiver operating characteristic curves for both models are shown in Figure 2.

**Fig. 3.**
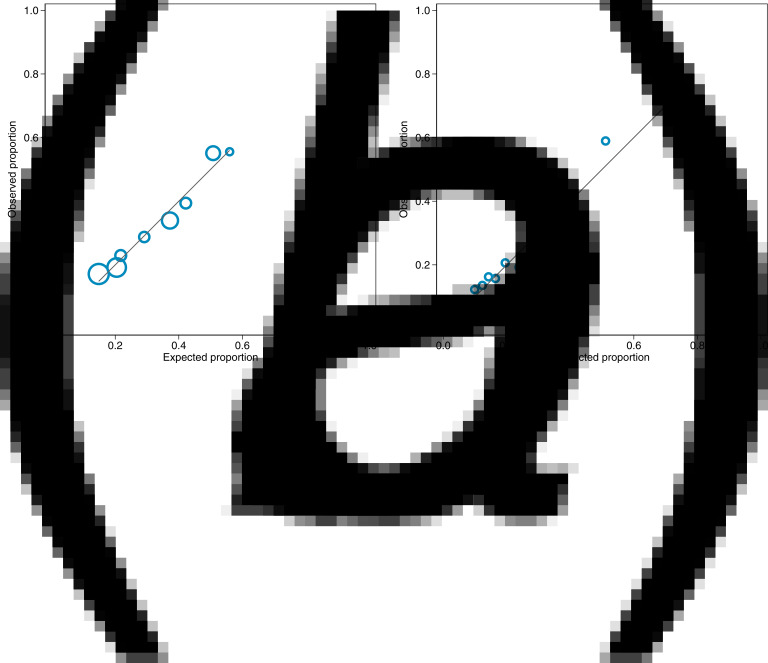
Calibration plots for the (a) age and sex model and (b) new model in the validation dataset.

Both models were well calibrated with no evidence of overall overestimation or underestimation for the age and sex model (*α* = −0.02; 95% CI −0.18 to 0.13; *P* = 0.7) or the new model (*α* = −0.08; 95% CI −0.21 to 0.05; *P* = 0.3). There was also no evidence of under or over dispersion for the age and sex model (*β* = 0.96, 95% CI 0.81–1.10, *P* = 0.6) and for the new model (*β* = 0.90, 95% CI 0.80–1.00, *P* = 0.06). Calibration plots for both models are shown in Figure 3.

#### Probability of severe COVID-19 in whole UK Biobank

**Fig. 4.**
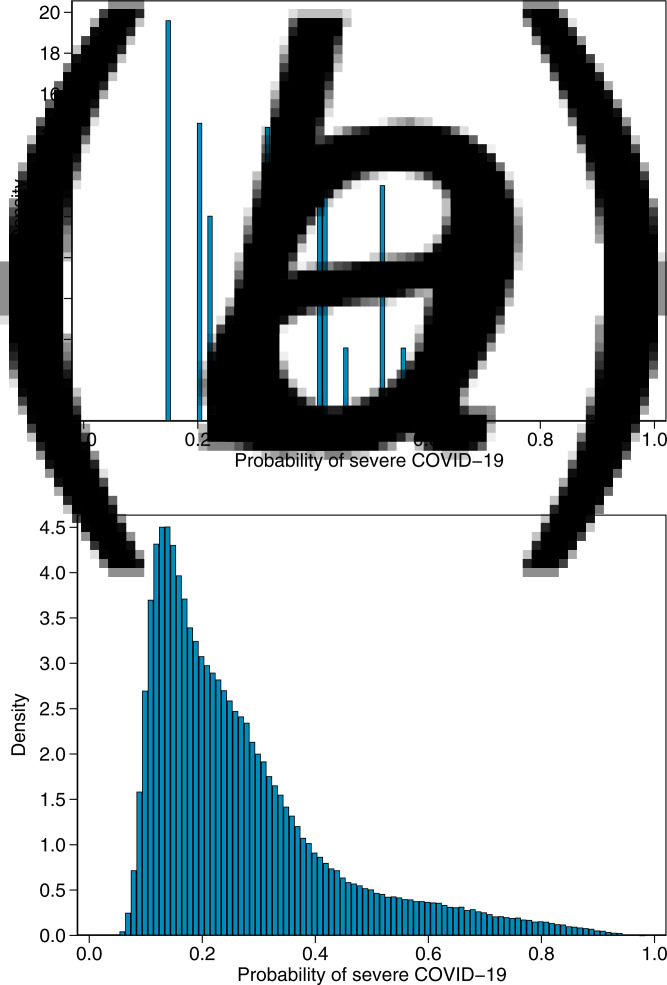
Distribution of probability of severe COVID-19 in all of UK Biobank for (a) the age and sex model and (b) the new model.

We calculated the probability of severe COVID-19 for all UK Biobank participants who met our eligibility criteria for this study; the distributions are shown in Figure 4, and the distribution of the new model by 5-year age group are shown in Supplementary Figure S1. Using the age and sex model, the mean probability was 0.32 (s.d. = 0.13) and ranged from a minimum of 0.15 to a maximum of 0.56. Using the new model, the mean probability was 0.27 (s.d. = 0.16) and the range was from 0.04 to 0.98, a much wider range than for the age and sex model.

## Discussion

An accurate test to predict risk of severe COVID-19 can inform prioritisation of vaccine doses to those most at risk [25] and will be useful in regions in which vaccination is not widespread enough to provide herd immunity (either through unavailability or vaccine hesitancy), if available vaccines are not effective against variants of SARS-CoV-2, or if available vaccines are not indicated for some people.

The validation of the clinical component of our prototype model confirmed that it performed well with good discrimination (AUC = 0.711), but overall, it overestimated risk. The SNP score component of the prototype model was not confirmed in the validation dataset and is likely due to the prototype model having been developed in dataset with a high prevalence of severe COVID-19.

Given the failure to confirm our prototype SNP-based risk score, we incorporated SNPs in the new model without relying on published summary statistics and without assumptions as to the identity of the risk allele. We included the SNPs as individual risk factors and estimated the per allele OR for each. By doing so, we were able to identify the subset of SNPs and clinical risk factors that were informative for predicting risk. These risk factors are all important to risk prediction, and characterisation of the SNP genotypes is as important as ascertaining clinical information.

From our initial list of 116 SNPs (Supplementary Table S1), we considered 14 for inclusion in our new model and retained seven, none of which were in the 3p21.31 locus identified by others [12–14, 22]. Three SNPs (rs35896106, rs76374459 and rs35652899) from the 3p21.31 locus had unadjusted associations with severe COVID-19 but these associations were better explained by the inclusion of the respiratory disease variable. Therefore, these three SNPs do not appear in our new model.

Functionally, most of the SNPs retained in our new model are associated with genes that play a role in infection pathways or immunity. The immune function and chromatin remodelling family of GATA transcription factors are associated by the inclusion of SNPs near *HIVEP1* (rs10755709), which encodes a viral-infection regulation transcription factor, and *GATA3* (rs71481792) [26, 27]. *ALPK1* and *TIFA* are closely downstream of rs112641600 and both have adaptive and innate signal transduction roles and pro-inflammatory functions [28]. *MSR1*, upstream of rs118072448, is a macrophage scavenger receptor and implicated in a broad range of disease types including host viral defence [29] and *PSAT1* is associated with glutamine metabolic reprogramming by SARS-CoV-2 and viral mRNA translation [30].

In the development of the new model, the strongest risk factor was respiratory disease (with an odds per standard deviation of 1.35; Table 3). The older age groups (70–74, 75–79 and 80+ years) and being male all had odds per standard deviations of 1.20–1.29. The other risk factors (the seven SPNs, ethnicity, BMI, cancer history (haematological and non-haematological), cerebrovascular disease, diabetes, hypertension and kidney disease) all had odds per adjusted standard deviations in the range 1.07–1.13 (or the equivalent protective effect).

In the non-imputed validation dataset, the new model performed very well with an AUC of 0.732 (compared with an AUC of 0.752 in the training dataset). Importantly, the new model was well calibrated, showing no evidence of problems with the overall estimation of risk or the dispersion of risk predictions. The validation of the new model also illustrates the importance of considering risk factors beyond age and sex in predicting risk of severe COVID-19. The new model was a substantial improvement over the age and sex model, in terms of the OR per quintile (OR 1.77 and OR 1.49, respectively) and the discrimination of cases and controls (AUC = 0.732 and AUC = 0.671, respectively). The new model also allows stratification across a wide range of risk (Fig. 4b) so that, for example, a healthy person aged 75 years might have a lower risk of severe COVID-19 than a 50-year-old person with several risk factors.

A limitation of this study is that, through necessity, we used hospitalisation as a proxy for COVID-19 severity and the outcome measure may have been misclassified for some participants. This would have attenuated the observed associations and it is possible that some risk factors have been omitted unnecessarily. Nevertheless, we are confident in the variables retained. We were unable to develop models for other important endpoints such as respiratory support, intensive care admission, or death because information on these were unavailable.

The progression of the COVID-19 pandemic has seen people experience chronic symptoms, and some of these people will have had only a mild original infection [5]. Identifying people who are at increased risk of chronic disease is an obvious direction for future research. Another direction for future research is to investigate whether our model for the prediction of severe COVID-19 is applicable for the new SARS-CoV-2 variants of concern, which have been reported to have increased transmissibility, virulence and antigenicity and cause more severe disease [3, 4]. Further validation of our new model is required in independent datasets, especially those in which the SARS-CoV-2 variant has been characterised.

Clear benefits of our new model for predicting risk of severe COVID-19 are that the required clinical data is simple to collect and that the genetic information is amenable to high-throughput genotyping, with rapid turnaround that is essential for the present pandemic. In the light of the uncertainty of the future of the COVID-19 pandemic, accurate knowledge of individual risk of severe COVID-19 can make an important contribution to healthcare on a population level and on a personal level.

## Supplementary material

For supplementary material accompanying this paper visit http://dx.doi.org/10.1017/S095026882100145X. click here to view supplementary material
