# Supplementary material for: Development and validation of a clinical and genetic model for predicting risk of severe COVID-19
Source: Epidemiol Infect. 2021 Jul 2;149:e162. doi: 10.1017/S095026882100145X (PMC8292840; doi:10.1017/S095026882100145X)
Supplement: Supplementary file 1 [file S095026882100145Xsup001.docx]

**Epidemiology and Infection**

# Development and validation of a clinical and genetic model for predicting risk of severe COVID-19

Gillian S. Dite^1*^

Nicholas M. Murphy^1^

Richard Allman^1^

## Supplementary figure

**Supplementary Figure S1. Distribution of probability of severe COVID-19 by 5-year age group.**

## Supplementary tables

**Supplementary Table S1. Allele frequencies and unadjusted odds ratios in the training dataset for the full list of SNPs identified as potential risk factors for severe COVID-19 (shaded SNPs were selected)**

| **Chr** | **SNP** | **Reference allele frequency** | | **Effect allele frequency** | | **OR** | **95% CI** | **P** |
| --- | --- | --- | --- | --- | --- | --- | --- | --- |
| **64 SNPs from Dite et al. [1]** | | | |  |  |  |  |  |
| 1 | rs12745140 | G | 0.91 | A | 0.09 | 0.90 | 0.77, 1.06 | 0.2 |
| 1 | rs12083278 | G | 0.29 | C | 0.71 | 1.05 | 0.96, 1.16 | 0.3 |
| 1 | rs2765013 | C | 0.91 | T | 0.09 | 1.10 | 0.96, 1.27 | 0.2 |
| 1 | rs2274122 | G | 0.20 | A | 0.80 | 0.97 | 0.88, 1.07 | 0.5 |
| 1 | rs10873821 | C | 0.75 | T | 0.25 | 0.92 | 0.84, 1.02 | 0.1 |
| 2 | rs6714112 | C | 0.86 | A | 0.14 | 1.03 | 0.91, 1.16 | 0.7 |
| 2 | rs2270360 | A | 0.74 | C | 0.26 | 0.94 | 0.85, 1.04 | 0.2 |
| 3 | rs1504061 | C | 0.95 | G | 0.05 | 1.13 | 0.94, 1.36 | 0.2 |
| 3 | rs17317135 | G | 0.95 | A | 0.05 | 0.89 | 0.73, 1.09 | 0.2 |
| 3 | rs1868132 | C | 0.90 | T | 0.10 | 1.02 | 0.89, 1.17 | 0.8 |
| 3 | rs6440031 | A | 0.08 | G | 0.92 | 0.95 | 0.80, 1.13 | 0.6 |
| 4 | rs3774881 | T | 0.84 | C | 0.16 | 0.91 | 0.82, 1.02 | 0.1 |
| 4 | rs3774882 | C | 0.92 | G | 0.08 | 0.89 | 0.76, 1.05 | 0.2 |
| 4 | rs6810404 | C | 0.51 | A | 0.49 | 0.97 | 0.89, 1.05 | 0.5 |
| 4 | rs35540967 | T | 0.93 | C | 0.07 | 1.01 | 0.87, 1.19 | 0.9 |
| 4 | rs115162070 | G | 0.93 | A | 0.07 | 0.90 | 0.75, 1.07 | 0.2 |
| 4 | rs11729561 | T | 0.92 | C | 0.08 | 0.96 | 0.82, 1.12 | 0.6 |
| 4 | rs112641600 | C | 0.89 | T | 0.11 | 0.83 | 0.72, 0.96 | 0.01 |
| 5 | rs62377777 | T | 0.79 | C | 0.21 | 0.96 | 0.87, 1.07 | 0.5 |
| 5 | rs4240376 | G | 0.80 | T | 0.20 | 0.98 | 0.88, 1.09 | 0.7 |
| 5 | rs10039856 | C | 0.90 | T | 0.10 | 1.10 | 0.96, 1.26 | 0.2 |
| 5 | rs2220543 | T | 0.71 | A | 0.29 | 1.03 | 0.94, 1.14 | 0.5 |
| 5 | rs113791144 | C | 0.93 | T | 0.07 | 0.97 | 0.82, 1.14 | 0.7 |
| 6 | rs6933436 | A | 0.71 | C | 0.29 | 1.01 | 0.92, 1.11 | 0.9 |
| 6 | rs10755709 | A | 0.70 | G | 0.30 | 1.11 | 1.01, 1.21 | 0.03 |
| 6 | rs140247774 | C | 0.93 | T | 0.07 | 0.93 | 0.78, 1.10 | 0.4 |
| 6 | rs16873740 | T | 0.88 | A | 0.12 | 1.16 | 1.03, 1.32 | 0.02 |
| 6 | rs9386484 | T | 0.76 | A | 0.24 | 0.95 | 0.85, 1.06 | 0.4 |
| 8 | rs118072448 | T | 0.92 | C | 0.08 | 0.82 | 0.70, 0.97 | 0.02 |
| 8 | rs10808999 | A | 0.13 | G | 0.87 | 1.01 | 0.89, 1.14 | 0.9 |
| 8 | rs13282163 | A | 0.92 | C | 0.08 | 0.93 | 0.80, 1.09 | 0.4 |
| 8 | rs11779911 | C | 0.67 | A | 0.33 | 0.99 | 0.91, 1.09 | 0.9 |
| 8 | rs2010843 | T | 0.47 | C | 0.53 | 1.04 | 0.96, 1.13 | 0.4 |
| 9 | rs3895472 | T | 0.08 | C | 0.92 | 1.03 | 0.88, 1.21 | 0.7 |
| 9 | rs12236000 | G | 0.92 | C | 0.08 | 0.95 | 0.81, 1.11 | 0.5 |
| 9 | rs7027911 | G | 0.57 | A | 0.43 | 1.10 | 1.01, 1.21 | 0.04 |
| 10 | rs71481792 | A | 0.38 | T | 0.62 | 0.89 | 0.82, 0.97 | 0.01 |
| 10 | rs2091431 | A | 0.28 | G | 0.72 | 1.04 | 0.94, 1.14 | 0.5 |
| 10 | rs1892429 | A | 0.84 | G | 0.16 | 0.98 | 0.87, 1.11 | 0.8 |
| 10 | rs10793436 | G | 0.68 | T | 0.32 | 0.94 | 0.85, 1.04 | 0.2 |
| 10 | rs1441121 | T | 0.57 | A | 0.43 | 0.95 | 0.87, 1.03 | 0.2 |
| 11 | rs10766439 | A | 0.37 | G | 0.63 | 0.97 | 0.89, 1.05 | 0.4 |
| 12 | rs11613792 | A | 0.85 | G | 0.15 | 1.01 | 0.89, 1.14 | 0.9 |
| 12 | rs12823094 | T | 0.76 | A | 0.24 | 1.08 | 0.98, 1.19 | 0.1 |
| 13 | rs1984162 | A | 0.75 | G | 0.25 | 1.10 | 1.00, 1.21 | 0.05 |
| 13 | rs12871414 | C | 0.74 | T | 0.26 | 0.95 | 0.86, 1.05 | 0.3 |
| 14 | rs2238187 | A | 0.65 | G | 0.35 | 1.07 | 0.98, 1.17 | 0.1 |
| 14 | rs12587980 | C | 0.63 | T | 0.37 | 1.03 | 0.94, 1.13 | 0.5 |
| 15 | rs12593288 | C | 0.80 | T | 0.20 | 0.91 | 0.82, 1.01 | 0.08 |
| 15 | rs2229117 | G | 0.86 | C | 0.14 | 0.90 | 0.80, 1.02 | 0.1 |
| 16 | rs72803978 | A | 0.94 | G | 0.06 | 0.88 | 0.74, 1.05 | 0.2 |
| 17 | rs34761447 | C | 0.90 | T | 0.10 | 1.02 | 0.89, 1.18 | 0.8 |
| 17 | rs178840 | G | 0.75 | A | 0.25 | 0.94 | 0.85, 1.04 | 0.2 |
| 18 | rs12958013 | T | 0.86 | C | 0.14 | 1.08 | 0.96, 1.22 | 0.2 |
| 19 | rs8105499 | C | 0.70 | A | 0.30 | 0.98 | 0.90, 1.08 | 0.7 |
| 19 | rs60744406 | A | 0.41 | G | 0.59 | 1.02 | 0.94, 1.11 | 0.7 |
| 19 | rs10411226 | G | 0.25 | A | 0.75 | 1.04 | 0.94, 1.15 | 0.5 |
| 21 | rs2252109 | A | 0.48 | T | 0.52 | 0.98 | 0.90, 1.06 | 0.6 |
| 22 | rs5757427 | T | 0.65 | A | 0.35 | 0.96 | 0.88, 1.05 | 0.4 |
| 22 | rs7290963 | G | 0.55 | T | 0.45 | 1.00 | 0.92, 1.09 | 0.9 |
| 22 | rs11090305 | T | 0.80 | C | 0.20 | 1.06 | 0.96, 1.18 | 0.2 |
| 22 | rs62220604 | G | 0.73 | A | 0.27 | 0.97 | 0.88, 1.07 | 0.5 |
| 3 | rs11385942 | G | 0.92 | GA | 0.08 | 1.16 | 1.00, 1.34 | 0.05 |
| 9 | rs657152 | C | 0.63 | A | 0.37 | 0.95 | 0.87, 1.03 | 0.2 |
| **12 SNPs from Pairo-Castineira et al. [2]** | | | |  |  |  |  |  |
| 3 | rs71325088 | T | 0.92 | C | 0.08 | 1.15 | 0.99, 1.33 | 0.07 |
| 3 | rs73064425 | C | 0.92 | T | 0.08 | 1.15 | 0.99, 1.33 | 0.07 |
| 6 | rs9380142 | G | 0.30 | A | 0.70 | 1.08 | 0.99, 1.19 | 0.09 |
| 6 | rs143334143 | G | 0.93 | A | 0.07 | 1.00 | 0.85, 1.18 | 1.0 |
| 6 | rs3131294 | A | 0.13 | G | 0.87 | 1.00 | 0.88, 1.13 | 1.0 |
| 12 | rs6489867 | C | 0.36 | T | 0.64 | 0.98 | 0.90, 1.07 | 0.7 |
| 12 | rs10735079 | G | 0.36 | A | 0.64 | 0.98 | 0.90, 1.07 | 0.6 |
| 19 | rs2109069 | G | 0.68 | A | 0.32 | 1.01 | 0.92, 1.11 | 0.8 |
| 19 | rs74956615 | T | 0.95 | A | 0.05 | 1.05 | 0.87, 1.29 | 0.6 |
| 19 | rs11085727 | C | 0.72 | T | 0.28 | 1.06 | 0.96, 1.16 | 0.2 |
| 21 | rs13050728 | C | 0.68 | T | 0.32 | 1.08 | 0.99, 1.18 | 0.1 |
| 21 | rs2236757 | G | 0.70 | A | 0.30 | 1.06 | 0.97, 1.16 | 0.2 |
| **40 SNPs identified from Host Genetics Initiative [3]** | | | | |  |  |  |  |
| 1 | rs17102023 | A | 1.00 | G | 0.00 | 1.03 | 0.62, 2.74 | 0.5 |
| 1 | rs115492982 | G | 1.00 | A | 0.00 | 2.46 | 1.23, 4.91 | 0.01 |
| 1 | rs2224986 | C | 0.91 | T | 0.09 | 0.98 | 0.85, 1.14 | 0.8 |
| 1 | rs74508649 | C | 1.00 | T | 0.00 | 1.05 | 0.47, 2.34 | 0.9 |
| 1 | rs112317747 | T | 0.97 | C | 0.03 | 1.26 | 1.00, 1.58 | 0.05 |
| 2 | rs183569214 | G | 1.00 | A | 0.00 | 0.71 | 0.15, 3.41 | 0.7 |
| 2 | rs77764981 | T | 1.00 | C | 0.00 | 1.29 | 0.54, 3.10 | 0.6 |
| 2 | rs2034831 | A | 0.94 | C | 0.06 | 1.23 | 1.04, 1.45 | 0.02 |
| 3 | rs1705826 | C | 0.63 | G | 0.37 | 1.03 | 0.94, 1.12 | 0.6 |
| 3 | rs35896106 | C | 0.92 | T | 0.08 | 1.17 | 1.01, 1.35 | 0.04 |
| 3 | rs76374459 | G | 0.94 | C | 0.06 | 1.21 | 1.02, 1.42 | 0.03 |
| 3 | rs35652899 | C | 0.93 | G | 0.07 | 1.17 | 1.01, 1.36 | 0.04 |
| 3 | rs12639224 | C | 0.73 | T | 0.27 | 1.02 | 0.93, 1.12 | 0.6 |
| 3 | rs34901975 | G | 0.89 | A | 0.11 | 1.11 | 0.98, 1.27 | 0.1 |
| 3 | rs71615437 | A | 0.92 | G | 0.08 | 1.11 | 0.96, 1.29 | 0.2 |
| 3 | rs13433997 | T | 0.88 | C | 0.12 | 1.10 | 0.97, 1.24 | 0.1 |
| 3 | rs10510749 | C | 0.91 | T | 0.09 | 0.99 | 0.85, 1.15 | 0.9 |
| 3 | rs115102354 | A | 0.95 | G | 0.05 | 0.96 | 0.79, 1.16 | 0.7 |
| 3 | rs13062942 | A | 0.64 | G | 0.36 | 0.93 | 0.85, 1.02 | 0.1 |
| 3 | rs76488148 | G | 0.96 | T | 0.04 | 1.25 | 1.02, 1.52 | 0.03 |
| 5 | rs4478338 | T | 0.92 | G | 0.08 | 1.08 | 0.93, 1.25 | 0.3 |
| 5 | rs111265173 | C | 1.00 | T | 0.00 | 0.98 | 0.35, 2.67 | 1.0 |
| 6 | rs61611950 | C | 0.99 | T | 0.01 | 0.92 | 0.56, 1.51 | 0.8 |
| 7 | rs6967210 | T | 0.99 | C | 0.01 | 1.16 | 0.84, 1.59 | 0.4 |
| 8 | rs332040 | G | 0.53 | A | 0.47 | 1.00 | 0.92, 1.09 | 0.9 |
| 9 | rs71480372 | A | 0.66 | T | 0.34 | 0.99 | 0.90, 1.09 | 0.8 |
| 9 | rs74790577 | A | 1.00 | T | 0.00 | 1.05 | 0.27, 4.06 | 0.9 |
| 10 | rs5016035 | T | 0.51 | G | 0.49 | 1.00 | 0.91, 1.09 | 0.9 |
| 12 | rs7397549 | T | 0.59 | C | 0.41 | 1.00 | 0.91, 1.09 | 0.9 |
| 13 | rs2649134 | C | 0.97 | T | 0.03 | 0.93 | 0.72, 1.19 | 0.6 |
| 14 | rs144114696 | G | 1.00 | A | 0.00 | 2.49 | 0.50, 12.38 | 0.3 |
| 15 | rs77055952 | A | 0.95 | G | 0.05 | 1.07 | 0.88, 1.29 | 0.5 |
| 15 | rs74750712 | T | 1.00 | G | 0.00 | 1.33 | 0.66, 2.70 | 0.4 |
| 16 | rs72779789 | G | 0.95 | C | 0.05 | 1.03 | 0.85, 1.26 | 0.8 |
| 16 | rs145643452 | G | 0.99 | A | 0.01 | 1.03 | 0.61, 1.74 | 0.9 |
| 17 | rs9890316 | G | 0.69 | A | 0.31 | 1.01 | 0.92, 1.11 | 0.9 |
| 18 | rs142257532 | T | 0.97 | C | 0.03 | 1.01 | 0.78, 1.30 | 1.0 |
| 20 | rs56259900 | A | 1.00 | G | 0.00 | 1.11 | 0.63, 1.94 | 0.7 |
| 20 | rs76253189 | C | 0.99 | G | 0.01 | 1.01 | 0.72, 1.42 | 1.0 |
| 21 | rs75994231 | C | 0.98 | T | 0.02 | 1.06 | 0.79, 1.43 | 0.7 |

Chr, chromosome number; CI, confidence interval; OR, odds ratio per effect allele

Note: imputed data was used for the unadjusted odds ratios; the 64 SNPs used in Dite et al. [1] included rs11385942 and rs657152 from Ellinghaus et al. [4]

1. **Dite GS, Murphy NM, Allman R.** (2021) An integrated clinical and genetic model for predicting risk of severe COVID-19: A population-based case–control study. *PLoS One*; **16**: e0247205.
2. **Pairo-Castineira E, et al.** (2021) Genetic mechanisms of critical illness in Covid-19. *Nature*; **591**: 92–98.
3. **COVID-19 Host Genetics Initiative**. COVID19hg GWAS meta-analyses round 4. (<https://www.covid19hg.org/results/r4/>). Accessed 11 December 2020.
4. **Ellinghaus D, et al.** (2020) Genomewide association study of severe Covid-19 with respiratory failure. *New England Journal of Medicine*; **383**: 1522–1534.

**Supplementary Table S2. Characteristics of cases and controls for validation of the prototype model to predict severe COVID-19**

| **Variable** |  | **Cases**  **N=1,234** | **Controls**  **N=4,805** |
| --- | --- | --- | --- |
|  |  | **Mean (SD)** | **Mean (SD)** |
| SNP score | % risk alleles | 61.3 (3.9) | 61.4 (3.8) |
|  |  | **N (%)** | **N (%)** |
| Age group (years) | 50–59 | 187 (15.2) | 1,717 (35.7) |
|  | 60–69 | 289 (23.4) | 1,609 (33.5) |
|  | 70+ | 758 (61.4) | 1,479 (30.8) |
| Sex | Female | 529 (42.9) | 2,613 (54.4) |
|  | Male | 705 (57.1) | 2,192 (45.6) |
| Ethnicity | White | 1,137 (92.1) | 4,433 (92.3) |
|  | Other | 87 (7.1) | 359 (7.5) |
|  | Missing | 10 (0.8) | 13 (0.3) |
| ABO blood type | O | 524 (42.5) | 1,855 (38.6) |
|  | A | 537 (43.5) | 2,244 (46.7) |
|  | B | 124 (10.1) | 504 (10.5) |
|  | AB | 49 (4.0) | 202 (4.2) |
| Autoimmune disease (rheumatoid arthritis, lupus or psoriasis) | No | 1,160 (94.0) | 4,635 (96.5) |
|  | Yes | 74 (6.0) | 170 (3.5) |
| Cancer – haematological | No | 1,206 (97.7) | 4,775 (99.4) |
|  | Yes | 28 (2.3) | 30 (0.6) |
| Cancer – non-haematological | No | 978 (79.3) | 4,227 (88.0) |
|  | Yes | 256 (20.8) | 578 (12.0) |
| Diabetes | No | 965 (78.2) | 4,393 (91.4) |
|  | Yes | 269 (21.8) | 412 (8.6) |
| Hypertension | No | 577 (46.8) | 3,428 (71.3) |
|  | Yes | 657 (53.2) | 1,377 (28.7) |
| Respiratory disease (excluding asthma) | No | 937 (75.9) | 4,456 (92.7) |
|  | Yes | 297 (24.1) | 349 (7.3) |

SD, standard deviation
